# Complete genome sequences of 37 bacteria in a maize rhizosphere synthetic community

**DOI:** 10.1128/mra.00496-25

**Published:** 2025-08-29

**Authors:** Ashley A. Paulsen, Dua X. Vang, Larry J. Halverson

**Affiliations:** 1 Department of Plant Pathology, Entomology, and Microbiology, Iowa State University 1177, Ames, Iowa, USA; 2 Ames National Laboratory, Iowa State University 17210, Ames, Iowa, USA; 3 Interdepartmental Genetics and Genomics Graduate Program, Iowa State University, Ames, Iowa, USA; 4 Interdepartmental Microbiology Graduate Program, Iowa State University, Ames, Iowa, USA; California State University San Marcos, San Marcos, California, USA

**Keywords:** synthetic bacterial community, rhizosphere-inhabiting microbes

## Abstract

We report the complete *de novo* genome assemblies of 37 bacteria isolated from soil and the maize rhizosphere and endosphere. These strains were selected for use in creating synthetic communities for studying the maize root microbiome.

## ANNOUNCEMENT

We created a maize rhizosphere bacterial synthetic community (MARSc) as a tool for studying maize rhizosphere microbiome assembly and for understanding interactions influencing rhizosphere colonization. Bacteria were isolated from the bulk soil, rhizosphere, and endosphere of V4-V5 Phz51 or BGEM-0111-S/Phz51 maize (1) grown in soil with low inorganic nitrogen inputs from the Marsden site (2) per protocol (3), and more than 400 isolates were screened for potential inclusion. Isolates were identified by sequencing 16S ribosomal RNA amplicons (V1-V9) and were selected for MARSc based on phylogeny, their interactions with other members, and maize rhizosphere relative abundance (2, 4). Here we present closed genomes for all members to further their usability as a tool for microbiology research.

For Illumina sequencing, DNA was isolated with the Wizard gDNA Isolation Kit (Promega). For ONT sequencing, high-molecular weight DNA was extracted using a modified phenol-chloroform-based protocol (5–7). Cultures were grown shaking overnight in 25 mL of media (Table 1) at 30° C; *Methylobacterium* was cultured in 50 mL due to slow growth. Extraction modifications include washing the cells with phosphate buffered saline and the use of phase-lock gel to limit pipetting. *Actinomycetota* isolates, especially *Streptomyces*, proved difficult to extract DNA from without mechanical lysis and required an optimized lysis protocol. Modifications included culturing with 0.5% glycine and the addition of 25% (w/v) sucrose to the lysis buffer (8, 9). A size-selective precipitation of fragments >30 kb was performed using a 9% polyethylene glycol 8 k (w/v), 1 M NaCl, 10 mM Tris-HCl (pH 8) buffer (10, 11). For post-size selection, DNA was homogenized per protocol (7), and fragment sizes were assessed using an Agilent 5200 Fragment Analyzer.

**TABLE 1:** Summary data of MARSc assemblies^*a*^

BioProject	Accession	MARSc ID	Organism	Cultured in	Illumina SRA	Illumina read counts	Fold short coverage	ONT SRA accessions no.	ONT read counts	ONT N50	Total fold long coverage	Assembly N50 (bp)	Genome size (bp)	Percent GC	Contigs	GenBank accession no.	Chromosome/chromid size (mb)	Plasmid size (kb)	Complete- ness	Contamination	Genes	CDS
PRJNA1228643	SAMN47200760	E-01	*Dyadobacter endophyticus*	1/2 TSB	SRR32597164	2,549,363	63	SRR32610437 SRR32610436 SRR32610425	35,769 79,978 4,343	19,453 3,561 47,076	56	7,769,076	7,769,076	49.6	1	CP183969	7.8		100	0.5	6,370	6,318
PRJNA1228643	SAMN47200761	E-04	*Agrobacterium radiobacter*	1/2 TSB	SRR32597163	2,156,582	68	SRR32610414	17,280	20,980	21	2,874,567	5,586,394	59.3	3	CP184251 CP184252 CP184253	2.9 2.2	536	100	0	5,344	5,273
PRJNA1228643	SAMN47200762	E-05	*Pseudomonas brassicacearum*	1/2 TSB	SRR32597152	1,581,558	44	SRR32610403	66,735	9,701	34	6,713,799	6,713,799	60.9	1	CP183970	6.7		98.8	3	6,028	5,942
PRJNA1228643	SAMN47200763	E-08	*Streptomyces* sp. E-08	1/2 TSB with 0.5% glycine	SRR32597141	2,142,866	49	SRR32610392 SRR32610386	7,949 258,059	19,349 9,245	159	8,612,121	8,612,121	72.4	1	CP183971	8.6		100	0.6	7,888	7,787
PRJNA1228643	SAMN47200764	E-12	*Paenibacillus silvae*	1/2 TSB	SRR32597136	1,869,109	53	SRR32610385	20,871	16,489	25	6,804,124	6,804,124	47.1	1	CP183972	6.8		100	1.6	5,944	5,800
PRJNA1228643	SAMN47200765	E-13	*Microbacterium* sp. E-13	1/2 TSB	SRR32597135	1,737,516	83	SRR32610384	14,405	21,853	34	4,096,171	4,096,171	69.7	1	CP183973	4.1		99.5	0.5	3,797	3,741
PRJNA1228643	SAMN47200766	E-15	*Ensifer adhaerens*	1/2 TSB	SRR32597134	1,752,747	37	SRR32610435 SRR32610434 SRR32610383	24,950 3,172 6,721	10,824 10,444 26,393	34	3,975,239	7,439,792	62.3	4	CP184365 CP184366 CP184367 CP184368	4.0 1.8 1.5	174	100	0.3	7,085	7,002
PRJNA1228643	SAMN47200767	E-16	*Cellulosimicrobium* sp. E-16	1/2 TSB with 0.5% glycine	SRR32597133	1,751,406	73	SRR32610433	95,958	13,891	173	4,594,192	4,594,192	74.1	1	CP183974	4.6		100	0	4,041	3,977
PRJNA1228643	SAMN47200768	E-22	*Luteibacter* sp. E-22	1/2 TSB	SRR32597132	1,517,986	69	SRR32610432 SRR32610431 SRR32610430 SRR32610387	3,284 238,722 38,815 95,362	22,460 969 1,058 9,281	120	4,276,699	4,276,699	65.3	1	CP183975	4.3		99.8	0	3,792	3,731
PRJNA1228643	SAMN47200769	E-23	*Lysobacter soli*	1/2 TSB	SRR32597131	1,271,863	63	SRR32610429	15,689	17,978	23	3,965,274	3,965,274	67.7	1	CP183976	4.0		99.9	1.1	3,633	3,573
PRJNA1228643	SAMN47200770	R-03	*Rhodococcus fascians*	1/2 TSB with 0.5% glycine	SRR32597162	4,422,942	411	SRR32610428	151,471	9,727	151	5,254,681	5,429,226	64.5	3	CP184369 CP184370 CP184371	5.3	92 82	99.7	1.6	5,172	5,110
PRJNA1228643	SAMN47200771	R-06	*Pedobacter* sp. R-06	1/2 TSB	SRR32597161	4,177,598	183	SRR32610427 SRR32610426	2,874 95,974	20,919 12,032	102	6,682,119	6,682,119	39.0	1	CP183977	6.7		100	1.3	5,487	5,414
PRJNA1228643	SAMN47200772	R-07	*Streptomyces* sp. R-07	1/2 TSB with 0.5% glycine	SRR32597160	2,347,015	53	SRR32610424 SRR32610423	13,734 62,266	20,711 17,346	88	8,650,038	8,650,038	72.1	1	CP183978	8.7		100	0.4	7,914	7,817
PRJNA1228643	SAMN47200773	R-11	*Arthrobacter* sp. R-11	1/2 TSB with 0.5% glycine	SRR32597159	3,931,800	259	SRR32610422 SRR32610421 SRR32610420	6,305 65,836 20,152	23,436 3,574 30,953	107	4,433,660	4,433,660	66.4	1	CP183979	4.4		99.4	0.8	4,055	3,982
PRJNA1228643	SAMN47200774	R-17	*Exiguobacterium* sp. R-17	1/2 TSB	SRR32597158	1,828,824	119	SRR32610419	56,923	11,741	91	3,000,203	3,197,202	46.6	2	CP184372 CP184373	3.0	197	100	0.2	3,303	3,203
PRJNA1228643	SAMN47200775	R-19	*Ensifer* sp. R-19	1/2 TSB	SRR32597157	1,758,207	43	SRR32610418 SRR32610417	3,859 37,289	26,118 17,434	55	4,024,228	7,208,945	62.0	3	CP184569 CP184570 CP184571	4.0 2.4	789	100	0	6,880	6,802
PRJNA1228643	SAMN47200776	R-20	*Methylobacterium fujisawaense*	R2A	SRR32597137 SRR32597156	1,197,906 4,335,750	210	SRR32610416	59,529	16,054	69	6,037,268	6,037,268	69.9	1	CP183980	6.0		100	0	5,717	5,645
PRJNA1228643	SAMN47200777	R-21	*Sphingobium* sp. R-21	1/2 TSB	SRR32597155	4,596,711	271	SRR32610415 SRR32610413 SRR32610412	5,182 29,177 2,626	24,754 15,540 47,731	48	3,078,074	4,816,019	63.2	3	CP184374 CP184375 CP184376	3.1 1.6	182	100	0.6	4,487	4,420
PRJNA1228643	SAMN47200778	R-24	*Stenotrophomonas maltophilia*	1/2 TSB	SRR32597154	2,161,962	92	SRR32610411	8,944	20,266	20	4,588,325	4,588,325	66.1	1	CP183981	4.6		100	0	4,181	4,090
PRJNA1228643	SAMN47200779	R-25	*Enterobacter ludwigii*	1/2 TSB	SRR32597153	1,768,633	72	SRR32610410 SRR32610409 SRR32610408	6,781 34,375 13,246	21,589 3,224 1,843	28	4,773,300	4,773,300	54.5	1	CP183982	4.8		99.9	0	4,562	4,445
PRJNA1228643	SAMN47200780	R-26	*Dyella kyungheensis*	1/2 TSB	SRR32597151	3,593,530	211	SRR32610407	14,028	21,800	29	4,969,812	4,969,812	64.2	1	CP183983	5.0		100	0.3	4,446	4,384
PRJNA1228643	SAMN47200781	R-27	*Variovorax* sp. R-27	1/2 TSB	SRR32597150	3,565,879	149	SRR32610406 SRR32610405	2,273 55,977	25,994 16,905	75	7,018,904	7,018,904	66.4	1	CP183984	7.0		100	0.3	6,604	6,538
PRJNA1228643	SAMN47200782	R-28	*Acidovorax facilis*	1/2 TSB	SRR32597149	3,595,431	193	SRR32610404 SRR32610402	689 23,087	26,222 16,832	38	5,486,923	5,486,923	64.4	1	CP183985	5.5		100	0.6	5,041	4,971
PRJNA1228643	SAMN47200783	R-29	*Ralstonia* sp. R-29	1/2 TSB	SRR32597148	4,953,628	253	SRR32610401 SRR32610400	8,155 351,080	21,020 5,600	177	3,653,769	6,031,623	63.7	4	CP184377 CP184378 CP184379 CP184380	3.7 2.0	227114	100	0.7	5,571	5,504
PRJNA1228643	SAMN47200784	R-30	*Burkholderia ambifaria*	1/2 TSB	SRR32597147	1,304,025	33	SRR32610399	22,774	17,440	22	2,774,643	7,467,149	66.7	3	CP184381 CP184382 CP184383	3.5 2.8 1.2		100	0	6,721	6,631
PRJNA1228643	SAMN47200785	R-31	*Pseudoduganella* sp. R-31	R2A	SRR32597146	5,628,344	242	SRR32610398	23,055	14,070	23	6,796,214	6,796,214	62.5	1	CP183986	6.8		100	0	6,194	6,097
PRJNA1228643	SAMN47200786	R-32	*Pseudoduganella* sp. R-32	R2A	SRR32597145	4,080,450	174	SRR32610397 SRR32610396 SRR32610395	24,476 246,749 67,188	14,559 8,563 13,698	282	6,869,375	6,869,375	62.5	1	CP183987	6.9		100	0	6,285	6,188
PRJNA1228643	SAMN47200787	R-34	*Pseudoduganella* sp. R-34	R2A	SRR32597144	3,036,901	131	SRR32610394	55,455	14,268	65	6,801,932	6,971,661	62.4	2	CP184572 CP184573	6.8	170	100	0	6,408	6,311
PRJNA1228643	SAMN47200788	R-43	*Pseudoduganella* sp. R-43	R2A	SRR32597143	3,809,374	164	SRR32610393	130,947	10,995	105	6,800,169	6,800,169	62.6	1	CP183988	6.8		100	0	6,209	6,112
PRJNA1228643	SAMN47200789	S-02	*Chryseobacterium* sp. S-02	1/2 TSB	SRR32597142	1,975,806	95	SRR32610391	16,675	22,961	42	4,059,545	4,059,545	34.9	1	CP183989	4.1		100	0	3,702	3,609
PRJNA1228643	SAMN47200790	S-10	*Micrococcus yunnanensis*	1/2 TSB	SRR32597140	2,218,458	174	SRR32610390	18,695	20,362	56	2,545,080	2,545,080	72.9	1	CP183990	2.5		99.9	0.2	2,380	2,322
PRJNA1228643	SAMN47200791	S-14	*Pseudoduganella* sp. S-14	R2A	SRR32597139	1,649,747	50	SRR32610389	51,972	12,318	59	6,430,702	6,430,702	62.5	1	CP183991	6.4		100	0	5,916	5,819
PRJNA1228643	SAMN47200792	S-18	*Bacillus velezensis*	1/2 TSB	SRR32597138	1,613,510	79	SRR32610388	25,153	18,361	50	3,976,484	3,976,484	46.5	1	CP184364	4.0		99.9	0.1	3,924	3,806
PRJNA1228643	SAMN48010940	R-33	*Mucilaginibacter* sp. R-33	1/2 TSB	SRR33160189	5,258,088	234	SRR33160179	147,543	9970	112	6,570,206	6,570,206	42.9	1	CP188089	6.6		99.8	0.6	5,504	5,437
PRJNA1228643	SAMN48010937	R-39	*Exiguobacterium* sp. R-39	1/2 TSB	SRR33160188	1,853,116	108	SRR33160184 SRR33160187	1,008,239 53,407	4721 7041	1094	3,085,405	3,166,508	47.0	2	CP187965 CP187966	3.1	81	100	0.1	3,293	3,193
PRJNA1228643	SAMN48010938	R-40	*Burkholderia ambifaria*	1/2 TSB	SRR33160186	1,239,526	33	SRR33160182 SRR33160183	6,605 71,926	17374 5356	37	2,805,731	7,484,842	66.6	3	CP187967 CP187968 CP187969	3.5 2.8 1.1		100	0	6,733	6,642
PRJNA1228643	SAMN48010939	S-38	*Paenibacillus* sp. S-38	1/2 TSB	SRR33160185	1,520,590	35	SRR33160180 SRR33160181	3,472 69,541	34357 32079	88	8,177,843	8,298,766	57.5	2	CP187970 CP187971	8.2	121	99.4	3.5	7321	7,116

^*a*^ The full table is available on FigShare at link:https://doi.org/10.6084/m9.figshare.29582999.v1.

All samples were sequenced using both Illumina and ONT. For Illumina, samples were sequenced either by the Iowa State University DNA Facility (HiSeq 3000) or by SeqCenter (NextSeq 2000); methods are listed in Table 1. All Illumina reads were trimmed and quality controlled using fastp v0.23.2 (12). For ONT, samples were sequenced on a GridION by the ISU DNA Facility, a PromethION by Plasmidsaurus, or a MinION Mk1C by our lab; methods are listed in Table 1. ONT barcodes and adapters were trimmed during demultiplexing with Dorado v0.5.1.

ONT reads were combined by strain and quality control was done using filtlong v.0.2.1 (–min_length 1000 –keep_percent 95) (13). Consensus assemblies were generated using Trycycler v.0.4.1 (14) and three different assembly programs: Flye v2.9 (15), Raven v1.6.1 (16), or Miniasm v0.3 (17) with Minipolish v0.1.3 (18). Additional programs were used for difficult-to-assemble strains (Table 1) (19–22). Assemblies were checked for over-circularization using self-versus-self alignments with MUMmer v3.23 (nucmer−maxmatch−nosimplify) (23) and terminal overlaps trimmed with BEDtools v2.27.1 “getfasta” (24). Assemblies were polished with ONT reads using Medaka v1.4.1 (ONT) and with Illumina reads using POLCA (MaSuRCA v4.0.5) (25) and PolyPolish v0.6.0 (26). All circular assemblies were rotated to start at *dnaA* or its equivalent using Circlator v1.5.5 “fixstart” (27). Linear contigs identified in species with known linear elements, notably *Streptomyces*, *Agrobacterium*, and *Rhodococcus*, were not rotated.

## Data Availability

This Whole Genome Shotgun project has been deposited in GenBank under the accession no. PRJNA1228643. Genome and Sequence Read Archive accession numbers are listed in Table 1. The code for the assembly pipeline is available on GitHub and Zenodo.
